# Drug Drop Test: How to Quickly Identify Potential Therapeutic Compounds for Mitochondrial Diseases Using Yeast *Saccharomyces cerevisiae*

**DOI:** 10.3390/ijms241310696

**Published:** 2023-06-27

**Authors:** Martina Magistrati, Alexandru Ionut Gilea, Maria Carla Gerra, Enrico Baruffini, Cristina Dallabona

**Affiliations:** Department of Chemistry, Life Sciences and Environmental Sustainability, University of Parma, Parco Area delle Scienze 11/A, 43124 Parma, Italy; martina.magistrati@unipr.it (M.M.); alexandruionut.gilea@unipr.it (A.I.G.); mariacarla.gerra@unipr.it (M.C.G.); enrico.baruffini@unipr.it (E.B.)

**Keywords:** drug drop test, yeast model, *Saccharomyces cerevisiae*, drug repurposing, mitochondrial diseases

## Abstract

Mitochondrial diseases (MDs) refer to a group of clinically and genetically heterogeneous pathologies characterized by defective mitochondrial function and energy production. Unfortunately, there is no effective treatment for most MDs, and current therapeutic management is limited to relieving symptoms. The yeast *Saccharomyces cerevisiae* has been efficiently used as a model organism to study mitochondria-related disorders thanks to its easy manipulation and well-known mitochondrial biogenesis and metabolism. It has been successfully exploited both to validate alleged pathogenic variants identified in patients and to discover potential beneficial molecules for their treatment. The so-called “drug drop test”, a phenotype-based high-throughput screening, especially if coupled with a drug repurposing approach, allows the identification of molecules with high translational potential in a cost-effective and time-saving manner. In addition to drug identification, *S. cerevisiae* can be used to point out the drug’s target or pathway. To date, drug drop tests have been successfully carried out for a variety of disease models, leading to very promising results. The most relevant aspect is that studies on more complex model organisms confirmed the effectiveness of the drugs, strengthening the results obtained in yeast and demonstrating the usefulness of this screening as a novel approach to revealing new therapeutic molecules for MDs.

## 1. Introduction

The budding yeast *Saccharomyces cerevisiae* is a single-cell eukaryotic microorganism commonly known as baker’s yeast. Despite its “simplicity”, it has strongly contributed to advancing our understanding of highly conserved basic cellular processes, such as cell cycle, vesicle trafficking, telomere function, mitochondrial function, autophagy, apoptosis, cytoskeletal dynamics, and aging [1]. Thanks to its contribution to our understanding of DNA repair in eukaryotes, yeast has even been defined as an “honorary mammal” [2]. Due to its similarities to higher eukaryotes, including humans [3], it has proven to be an excellent and powerful model organism to study fundamental aspects of complex eukaryotic organisms’ cell biology and cellular physiology related to human health and disease. Several cellular mechanisms are conserved between yeast and humans, including DNA replication, recombination, and repair; RNA transcription and translation; trafficking; metabolism; and, of particular relevance for this review, mitochondrial biogenesis [4]. *S. cerevisiae* is also an ideal organism for laboratory purposes. Its ability to grow quickly on simple media and in a huge variety of conditions makes studies of this organism fast and economical. Another important feature is that *S. cerevisiae* exists in both haploid and diploid states, leading to the possibility of identifying dominant and recessive mutations; in addition, genetic knockouts using simple methods can be produced, taking advantage of its compact genome and the efficient homologous recombination mechanism [5,6,7]. Beyond genomic manipulation, a variety of different plasmids are available, making it easy to introduce wild-type or mutant genes into the cell through simple transformation protocols. In addition, with the exception of *Chlamydomonas reinhardtii* [8], *S. cerevisiae* is the only eukaryote in which highly efficient biolistic transformation has been achieved [9] and site-directed mutagenesis of the mitochondrial genome has been established [10]. *S. cerevisiae* is the first eukaryote whose genome was entirely sequenced; the nucleotide sequence is 12.8 Mb, about 250 times shorter than that of the human genome [11]. A total of 31% of protein-coding genes in yeast possess a human orthologue, and about 46% of the known human protein-coding genes present a homolog in yeast [12], including proteins involved in mitochondrial biogenesis. For this reason, yeast has been widely used to validate and study mutations associated with mitochondrial dysfunction [13]. To study mitochondrial deficiencies, the ability of yeast, *S. cerevisiae,* to survive in the absence of respiratory functions can be exploited. Yeast metabolism depends on the carbon source added to the growth medium. In particular, in the presence of glucose, yeast preferentially performs glycolysis and alcoholic fermentation, while respiration is mostly suppressed; in contrast, in the presence of non-fermentable carbon sources, such as acetate, ethanol, glycerol, and lactate, yeast performs oxidative phosphorylation (OXPHOS) to produce ATP. In the latter case, mitochondrial functionality is strictly required; therefore, this growth condition can be exploited to identify mitochondrial dysfunction easily and quickly. In fact, when a mutation altering mitochondrial functionality occurs, a partial reduction or complete inhibition of oxidative growth can be observed.

Mitochondrial diseases (MDs) refer to a group of different clinical conditions whose common feature is defective mitochondrial function and energy production [14]. Mitochondrial disorders can be caused by mutations in either the nuclear or mitochondrial genome and are characterized by high genetic heterogeneity, making the diagnosis challenging. Although each individual disease is extremely rare, with only a handful of patients affected, when considered together, MDs are the most common group of inborn errors of metabolism, with a collective incidence of 1.6 in 5000 [15,16]. The clinical, biochemical, and molecular heterogeneity of the mitochondrial disease syndromes has hampered the development of therapeutic interventions. There is no effective treatment for most MDs, and current therapeutic management is limited to symptomatic relief and does not significantly alter the course of the disease [17,18]. Given that the currently available pharmacological intervention is inadequate, the identification of molecules with positive effects on mitochondrial functions in experimental models could be the first step toward an effective pharmacological therapy. Recently, yeast has been exploited to develop phenotype-based screenings for the discovery of potentially beneficial molecules able to rescue OXPHOS phenotypes associated with mitochondrial dysfunctions. *De novo* drug discovery is a long and complex process that takes many years to identify new molecules with a beneficial effect; to overcome this issue, a drug repurposing approach can significantly reduce time and costs [19]. Drug repurposing is defined as a process to find new therapeutic uses for approved drugs outside the scope of the original medical indication [20], meaning that we can take advantage of the off-label effects. The rationale is based on the fact that many drugs, in addition to the primary, usually known target, also have secondary, usually unknown off-targets; alternatively, the primary target could be directly or indirectly involved in a pathway embroiled in the onset of the disease. Combining the principles of drug repurposing and phenotype-based screening, it is possible to perform a screening of FDA-approved molecules, accelerating the drug discovery process by identifying new clinical uses for drugs already on the market. Among the high-throughput screening methods, a reverse halo laboratory assay called the drug drop test, which relies on the recovery of growth defects in the presence of a beneficial molecule, has been developed. The method was first described by Bach and collaborators, leading to the identification of anti-prion drugs in yeast that have shown efficacy on different mammalian systems [21,22], and was adapted for the study of mitochondrial dysfunctions by Couplan and colleagues [23]. In this context, the first drug drop test was performed in order to discover active compounds against NARP syndrome, a mitochondrial disease caused by a deficiency in ATP synthase assembly [23]. Yeast mutants defective in oxidative growth due to a defect in oxidative phosphorylation are initially spread on a solid medium containing a non-fermentable carbon source, and effective compounds are identified based on the presence of an enhanced halo of growth around the filters [23]. The drug drop test was also exploited to identify molecules to treat pathologies different from mitochondrial ones, adapting the growth conditions for each specific mutant phenotype. For example, yeast was successfully used to identify compounds for VPS13-dependent neurodegenerative diseases and GDAP1-dependent Charcot Marie Tooth (reviewed in [24]) [25].

In this review, we will focus on drug drop tests for the identification of potential therapeutic compounds to treat mitochondrial diseases and on the characterization of the drugs through different analyses, exploiting yeast *S. cerevisiae* as a model system. The models described here are summarized in Table 1.

## 2. The Drug Drop Test Method

**Figure 1:**
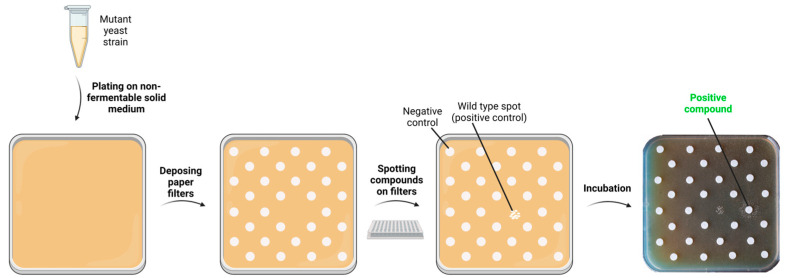
The drug drop test method. The mutant yeast strain is spread on square plates containing solid medium supplemented with a non-fermentable carbon source that allows basal minimal growth. Subsequently, small sterile filters are placed on the agar surface and spotted with molecules belonging to chemical libraries. In addition, negative and positive controls (i.e., the solvent in which molecules are dissolved and a wild-type spot, respectively) are deposed. The plate is then incubated for several days. After incubation, the positive compounds (active compounds) determine the appearance of a halo of enhanced growth around the corresponding filter. Created with BioRender.com, accessed on 29 May 2023.

Yeast mutant cells defective in oxidative growth are spread at a proper concentration on square plates containing solid media supplemented with a non-fermentable carbon source, where they grow minimally, and, afterward, sterile filters are placed on the agar surface and spotted with the compounds to test. One filter is spotted with the solvent in which the molecules are solubilized as a negative control; in addition, wild type (wt) and/or a known positive compound can be spotted as positive controls of growth. The plates are then incubated for several days until a difference in growth between the positive control and the mutant strain is clearly observed. Depending on the presence or absence of a halo of growth and its position around the filters, the compounds are classified into different groups: (i) compounds with a rescuing effect that lead to the formation of a halo of enhanced growth near the filter; (ii) compounds toxic at high concentrations (near the filter) and active at lower concentrations (far from the filter), which generate an external crown of enhanced growth; (iii) compounds without any effect if no enhanced growth is observed; and (iv) toxic compounds that completely inhibit the basal growth of the mutant strain. The diffusion of the molecule around the filter in the agar medium creates a concentration gradient, making it unnecessary to find the specific optimal drug concentration by testing several dilutions of every compound. Primary screening allows us, in one simple experiment, to test a huge number of drugs across a large range of concentrations thanks to their diffusion. Only a few extremely toxic molecules should be diluted and further tested. To confirm the rescue effect observed during primary screening and to minimize the number of false-positive molecules, the active compounds (positive compounds) are subjected to a second step of analysis performed using the same conditions, except for the number of filters placed on each plate, which decreases from about 30 to 4 (Figure 1).

## 3. Drug Drop Test for the Identification of Therapeutic Molecules for the Treatment of Mitochondrial Diseases

### 3.1. Identification of Molecules for the Treatment of ATP Synthase-Related Disorders

Impairment of ATP synthase (complex V), the enzyme responsible for the mitochondrial synthesis of ATP and the energy-carrying molecule used to power many energy-requiring cellular reactions, determines a group of mitochondrial disorders. The ATP synthase-related disorders are due to defects in the structure or in the assembly of complex V and include Leigh syndrome (fatal encephalopathy, OMIM #256000), hypertrophic cardiomyopathy, and NARP (neuropathy, ataxia, and retinitis pigmentosa, OMIM #551500) [35,36,37]. Mutations in the mitochondrial gene ATP6 (encoding ATP synthase subunit 6) and in the nuclear genes ATP12 (also known as ATPAF2, encoding ATP synthase mitochondrial F1 complex assembly factor 2) and TMEM70 (encoding the transmembrane protein 70) have been found in patients presenting ATP synthase deficiencies [38,39,40].

ATP6 is encoded by nucleotides 8527–9207 of the mitochondrial genome and, together with ATP8, make up the only mitochondrial ATP synthase subunits encoded by the mtDNA. Several mutations in ATP6 have been reported as causes of mitochondrial impairment. T8993G transversion was identified in a patient presenting mental retardation and ataxia without retinitis pigmentosa [41] and was later identified in other patients, as was T8993C transition [42,43]. The T8528C transition was identified in four unrelated infants presenting isolated hypertrophic cardiomyopathy and congestive heart failure that developed into severe feeding difficulties and failure to thrive [44]. Burrage and collaborators also identified the G8969A transition in a patient presenting mitochondrial myopathy, lactic acidosis, and sideroblastic anemia [45,46]. In addition, a patient carrying T9101C transition presented peripapillary microangiopathy [47], and several other patients have been reported with T9176C transition [48,49,50]. Substitutions T8851C [51], T9185C [52], T9205A [53], T8909C [54], T9191C [55], and the insertion of a thymine in position 8618 [56] have been reported through the years.

Due to the high number of mutations identified in this gene and, more generally, related to ATP synthase deficiencies, it is important to identify new therapeutic approaches to treat the correlated syndromes. The conservation of mitochondrial functions and the capacity to manipulate both mitochondrial and nuclear genomes in yeast allowed us to largely study in this organism the consequences of ATP synthase dysfunctions, whose pathogenic mechanisms could not be ascribed to a mere lack or decrease in ATP production [36].

Thanks to the biolistic technique, through which site-direct mutagenesis of the *S. cerevisiae* mitochondrial genome is possible, yeast models for the most common ATP6 mutations found in NARP patients have been created and characterized [46,54,55,57,58,59,60,61]. Another relevant yeast model for primary ATP synthase dysfunctions is represented by the *fmc1Δ* strain, even if humans do not present an orthologue of this gene. *FMC1* encodes a mitochondrial matrix protein required for the assembly and stabilization at high temperatures of the F1 subunit of the ATP synthase [62]. The null mutant (*fmc1Δ*) is thermosensitive and is characterized by a strong reduction in assembled ATP synthase complexes. Nevertheless, the few ones present are fully functional. A similar condition was also found in patients carrying mutations in the nuclear genes ATP12 and TMEM70 and in patients heteroplasmic for ATP6 mutations, indicating that the null mutant *fmc1Δ* represents a suitable model to study human ATP synthase dysfunctions, regardless of the specific genetic cause. As a secondary effect, the loss of *FMC1* negatively impacts respiratory chain complex assembly and activity and the mitochondrial membrane potential [63].

Couplan and colleagues developed the drug drop test to find compounds active against ATP synthase-related disorders, exploiting the leaky oxidative growth defect of the *fmc1Δ* strain on a glycerol-containing medium at 35 °C [23]. Around 12,000 compounds from different chemical libraries, including the Prestwick Chemical Library, containing drugs for which bioavailability and toxicity are known, were tested to evaluate their ability to rescue the growth defect. In addition, two molecules, dihydrolipoic acid (DHLA) and oleic acid (OA), were rationally selected. DHLA was previously used for the treatment of patients affected by mitochondrial encephalopathies [64,65]. The fatty acid OA was used as it is known to induce the expression of the Odc1 protein, which was previously reported as a multicopy suppressor of the OXPHOS defect of the *fmc1Δ* mutant [66,67]. Both molecules showed their ability to partially rescue, in a dose-dependent manner, the growth defect of the *fmc1Δ* strain and were used as positive controls during the high-throughput screening. In the primary screening, about ten molecules were identified as positive hits, among which was Chlorhexidine (CH), an antiseptic compound. CH was further tested on *atp6*-NARP models, showing a beneficial effect on the *atp6*-NARP T8993G and the *atp6*-NARP T8851C strains but not on the *atp6*-NARP T9176G strain, carrying the most severe mutation. This result could be due to the presence of a partially functioning ATP synthase in the former strains, so that the molecule could exert its beneficial effect [61]. To deepen our knowledge about the mechanism underlying the rescuing effect, oxygen consumption rates, respiratory enzyme abundance, energization of the mitochondrial membrane, ATP synthesis rates, assembly of ATP synthase, and mitochondrial morphology were evaluated. In particular, treatment with CH determined a partial restoration of the respiratory activity and steady-state levels of complex III-V subunits, a complete restoration of the energization of the mitochondrial membrane, a modest but significant increase in ATP synthesis rate, and a significant increase in the fully assembled ATP synthase amount. Regarding mitochondrial morphology, the typical defects of the *fmc1Δ* strain, such as the presence of inclusion bodies and the absence of mitochondrial *cristae*, were partially rescued after CH treatment. To get insight into the effect of the CH treatment on cellular function, genome-wide analyses of the transcriptional responses were performed through high-resolution tiling microarrays. A total of 336 genes, mostly involved in mitochondrial respiration, showed significant differential expression in the *fmc1Δ* mutant, and in almost all cases, treatment with chlorhexidine determined the rescue of the transcriptional differences, although to a different extent for the different genes. Overall, the data suggested that CH improved the respiratory function of the *fmc1Δ* null mutant. In particular, it was proposed that CH could act both by increasing the number of ATP synthase complexes and the efficiency with which they are used, resulting in a modest but significant increase in ATP production that in turn leads to the rescue of the oxidative growth defect [23].

To prove a possible translational use, the drugs identified through the yeast-based screening were also tested on human NARP-cybrids (cytoplasmic hybrids), used as human cell-based models. The results confirmed and strengthened the data obtained in yeast; in fact, DHLA, OA, and CH increased the growth rates of *atp6-*T8993G-NARP cybrids, for which a severe growth defect was previously reported [68]. The results suggested that the positive molecules DHLA, OA, and CH are able to improve ATP production through oxidative phosphorylation. Even if the increase in ATP is modest, it should be considered that small differences could be sufficient to restore a healthy state due to the threshold effect [69,70]. Furthermore, the positive molecules could induce the metabolic shift from glycolysis to OXPHOS. This is particularly valuable since one of the main symptoms in patients is lactic acidosis, probably due to glycolytic metabolism.

In a second screening based on the same approach (drug repurposing and drug drop test) and on the same yeast mutant, Aiyar and colleagues identified the antiseptic Sodium pyrithione (NaPT) as able to rescue in a dose-dependent manner the oxidative growth defect of the *fmc1Δ* strain [26]. This compound is also effective on *atp6-*T8993G-NARP cybrids [36,65], suggesting that NaPT restores ATP synthase deficiency through a pathway conserved between yeast and humans [26]. By using the yeast genome-wide deletion collection, systematic chemical-genomic profiling was used to identify the NaPT target pathways. The mutants mostly affected by inhibitory concentrations of NaPT are involved in mitochondrial protein sorting or in iron-sulfur cluster biogenesis pathways [71]. Regarding protein sorting, the heterozygous deletion mutants of *TIM17* and *TIM23* were identified. It was suggested that NaPT selectively and differentially modulates the import of proteins carrying pre-sequence signals through the Tim23 pathway. Supplementation of NaPT exerts a similar effect to those determined by *TIM21* overexpression, a regulatory subunit of the Tim23 complex, leading to a change in Tim23 activity from matrix translocation towards inner membrane insertion [72,73]. The mitochondrial protein import, due to its connection to complex assembly, was highlighted as an important intervention point since *TIM21* overexpression improved respiratory chain complex assembly. Furthermore, it was demonstrated that the modulation of mitochondrial protein sorting alleviates not only primary ATP synthase deficiency but also several downstream-related phenotypes [26,39,40,61,72,74].

Similar to what was observed in yeast, overexpression of TIM21 in human cells led to a significant increase in the survival of *atp6*-T8993G NARP patient-derived cybrids [26], underlying the conservation of the effect.

### 3.2. Identification of Molecules for the Treatment of ANT1-Related Disorders

ANT1, also known as SLC25A4, encodes the adenine nucleotide translocator 1, which is one of the four isoforms of the mitochondrial ADP/ATP carrier. It is located in the inner mitochondrial membrane and is involved in the import of ADP into the mitochondrial matrix, where its conversion to ATP by the ATP synthase occurs. Subsequently, it is also involved in the transport of the newly produced ATP to the cytosol [75]. Although the primary function of ANT1 is fully understood, its role in several other cellular processes, such as mtDNA maintenance [76], apoptosis [77], and mitophagy [78], has not yet been completely elucidated.

The first disease identified as being associated with ANT1 mutations was autosomal dominant progressive external ophthalmoplegia (adPEO; OMIM #609283), characterized by multiple mitochondrial DNA deletions in skeletal muscle. The most common clinical features include the adult onset of weakness of the external eye muscles, ptosis, myopathy, and exercise intolerance [79]. Pathogenic mutations causing adPEO were also found in POLG (encoding the mitochondrial polymerase [80]), POLG2 (encoding the POLG accessory subunit [81]), RRM2B (encoding the small subunit of p53-inducible ribonucleotide reductase [82]), TWNK (encoding the mitochondrial helicase Twinkle [83]), DNA2 (encoding the DNA replication helicase/nuclease 2 [84]), and indeed ANT1 [76].

To date, several degenerative mitochondrial pathologies caused by mutations in ANT1 are known; in particular, dominant missense point mutations (p.Ala90Asp, p.Leu98Pro, p.Asp104Gly, p.Ala114Pro, and p.Val289Met) were identified in subjects affected by adult-onset adPEO [76,85,86,87,88], while recessive missense point mutations (p.Ala123Asp and p.Arg236Pro) were identified in patients affected by mitochondrial myopathy and cardiomyopathy [89,90]. Furthermore, additional de novo dominant missense mutations, p.Arg80His and p.Arg235Gly, were identified in patients with severe congenital hypotonia and profound muscle weakness [91], and p.Lys33Gln was found in a patient with mild childhood-onset myopathy [92]. Despite the great differences in clinical presentations, deletions or depletions of mtDNA were found in the tissues of all affected patients [93,94,95], highlighting the protein involvement in mtDNA metabolism. One of the most challenging unsolved questions is how exactly the protein contributes to mtDNA stability maintenance. In fact, the pathogenic mechanism by which mutations in this gene are linked with mitochondrial genome defects remains to be elucidated.

Due to the lack of expression of ANT1 in cultured human cell lines, such as fibroblasts and myoblasts, the impact of ANT1 mutations could not be studied in these cells [96]. Studies on the pathogenic mechanism of ANT1 mutations were mostly carried out in the yeast *Saccharomyces cerevisiae*, thanks to the high conservation of the genes encoding the ADP/ATP carrier in eukaryotes, including yeast. Yeast has three genes that are orthologous to ANT1: *AAC1*, *AAC2*, and *AAC3*. *AAC2* encodes for the major isoform of the translocator [97], whereas Aac1 and Aac3 have a minor role in ADP/ATP translocation [98]. In particular, Aac2 is involved in oxidative phosphorylation and is required for growth on non-fermentable carbon sources, such as ethanol, glycerol, or lactate. Several mutations identified in ANT1 have been studied over the years through homologous complementation or chimeric complementation in *S. cerevisiae* by using the *aac2Δaac1Δ* strains carrying the human equivalent mutations in *AAC2* or in the chimeric gene [76,79,89,91,99,100]. In particular, the p.Met114Pro mutation was introduced in haploid yeast, creating the strain *aac2^M114P^*, to reproduce the p.Leu98Pro variant found in patients suffering from adPEO. The strain, characterized by severely affected but not completely abolished oxidative growth, was subjected to drug drop test screening to find molecules able to rescue the phenotype in yeast and therefore of potential therapeutic interest for the treatment of ANT1-related disorders [27]. Different oxidative carbon sources and cells per plate were tested to establish suitable conditions. The so-called primary screening was thus performed using acetate as a carbon source and 4.5 × 10^5^ cells/plate to test the 1018 molecules from the Selleck FDA-approved chemical library. Positive compounds that induce a halo of enhanced growth around the corresponding filter were identified after 3–4 days of incubation at 28 °C. Eight active molecules (doxorubicin, epirubicin HCl, daunorubicin HCl, otilonium bromide, trifluoperazine 2HCl, pergolide mesylate, sertraline HCl, and benzydamine HCl) confirmed their positive effect in the secondary screening. Drugs used for chemotherapy, such as doxorubicin, epirubicin, and daunorubicin, are not suitable for long-term treatment of mitochondrial pathologies and therefore are not used for further characterization; despite this, they may lead to the identification of potential molecular targets or pathways targetable by non-toxic analogous molecules. The other five drugs were also able to act on two additional *aac2* mutant strains characterized by a leaky oxidative growth defect, namely the strains presenting the mutations p.Ala128Pro and p.Ser303Met, corresponding to the human pathological mutations p.Ala114Pro and p.Val289Met, respectively. In contrast, the molecules were inactive in two mutant strains characterized by a strong oxidative growth defect and carrying p.Arg96His and p.Arg252Gly mutations, equivalent to the severe human dominant mutations p.Arg80His and p.Arg235Gly, respectively. The molecules were also inactive in the null mutant, indicating that the drugs were unable to bypass the Aac2 function.

To deepen our understanding of the mechanism underlying the beneficial effect, the five effective drugs were also evaluated on different defective phenotypes of the haploid mutant strain *aac2^M114P^*. They revealed a beneficial effect on respiratory activity but no beneficial action on mitochondrial membrane potential or the production of reactive oxygen species. Otilonium bromide showed a decoupling action, suggesting that the rescuing ability could be due to its capacity to reduce the electrochemical gradient across the inner mitochondrial membrane. Regarding the other drugs, the authors have speculated on a possible action on mitochondrial inner membrane lipid composition that could correct the proteostatic stress of the inner mitochondrial membrane (IMM) or the induction of an increase in Ca^2+^ levels that could, in turn, stimulate ATP-Mg/Pi carrier Sal1 transport activity [27].

In addition, Western blot analysis excluded the possibility that the treatment with molecules leads to an increase in Aac2 protein synthesis or to a stabilization of the protein. As the majority of ANT1 pathological mutations are dominant, heteroallelic strains could be used as a better model system to mimic the human heterozygous condition. Heteroallelic *AAC2/aac2^M114P^*, the model for the adPEO-associated human mutations p.Leu98Pro, and heteroallelic *AAC2/aac2^R96H^*, the model for the p.Arg80His *de novo* dominant missense mutation associated with a more severe phenotype, were used to assay the effect of the molecules. The drugs restored respiratory activity and reduced in both models the *petite* percentage, an index of mtDNA instability [27], which is observed in all patients affected by ANT1 mutations [76], thus expanding the possible applications for the treatment [76].

Moreover, studies on *C. elegans* ant1-1 by means of RNA interference demonstrated that the two tested drugs, otilonium bromide and pergolide mesylate, were able to decrease embryonic lethality, a worm-specific defect [27]. This observation gives further confirmation of the robustness and reliability of the results obtained in yeast and indicates that the drugs can also be active in a multi-organ animal model and thus potentially be applied to higher organisms.

### 3.3. Identification of Molecules for the Treatment of BCS1L-Related Disorders

BCS1L is the homolog of *S. cerevisiae BCS1*, a gene involved in complex III assembly of the mitochondrial respiratory chain; it encodes a protein involved in the incorporation of the nuclearly-encoded Rieske subunit (called UQCRFS1 in humans and Rip1 in yeast) in complex III, also known as the cytochrome bc_1_ complex. Mutations in this gene are the most common cause of complex III-related diseases, such as Bjornstad syndrome, GRACILE syndrome, and mitochondrial complex III deficiency type 1 (reviewed in [101]). Bjornstad syndrome (OMIM #262000) is an autosomal recessive disorder characterized by congenital sensorineural hearing loss and childhood-onset pili torti [102,103]. GRACILE syndrome (growth retardation, aminoaciduria, cholestasis, iron overload, lactic acidosis, early death, OMIM #603358) is a neonatal metabolic disorder characterized by severe intrauterine growth retardation, Fanconi-type aminoaciduria, and impairment of iron metabolism [104]. Mitochondrial complex III deficiency type 1 (OMIM #124000) is an autosomal recessive multisystem disorder characterized by a birth-onset of lactic acidosis, hypoglycemia, hypotonia, encephalopathy, failure to thrive, and delayed psychomotor development; death often occurs during childhood [105,106].

In *S. cerevisiae*, the impairment of Bcs1 determines the accumulation of an inactive form of complex III, pre-complex III (Pre-III), lacking Rip1, blocking the supercomplex III_2_-IV formation. Due to the dual genetic origin, the assembly of all the subunits into the mature and functional cytochrome bc_1_ complex is a complicated process that was largely studied in yeast (reviewed in [107]) and, despite some differences, is essentially conserved between yeast and humans [108]. Thanks to the high Bcs1 protein sequence conservation and the conserved Complex III assembly pathway, *S. cerevisiae* was used as a model system to study mutations identified in patients. Furthermore, it was demonstrated that the human BCS1L cDNA can complement the respiratory deficiency of the yeast null mutant [105], confirming the protein function conservation between yeast and humans.

In order to identify chemical compounds able to compensate for the respiratory deficiency in *bcs1* mutants, Panozzo and collaborators performed a drug drop test [28]. In particular, the Prestwick and the BIOLMOL chemical libraries, including a total of about 1600 molecules, were tested on the leaky *bcs1^F342C^* strain (carrying the substitution p.Phe342Cys) [28,109]. The *bcs1^F342C^* strain, characterized by a mutation located in the AAA domain of the protein, was spread on plates containing glycerol, where the respiratory defect was visible, and incubated for several days at 25 °C. Two compounds, the antibiotics pentamidine and clarithromycin, were able to partially rescue the defect. To test the potential effectiveness of these molecules for the treatment of patients, they were tested on *bcs1^K192P^*, mimicking the human p.Arg155Pro mutation, located in a Bcs1-specific domain and found in a patient presenting liver failure [105], and on *bcs1^F401I^*, mimicking the human p.Phe368Ile mutation, located in the AAA-domain and identified in a patient presenting early-onset encephalopathy [110]. The molecules showed a beneficial effect on the latter but not on the former, suggesting that the antibiotics can restore defects caused by mutations that strongly but not fully affect the activity of the protein. Moreover, the two antibiotics were tested on *bcs1Δ*, showing no effect, indicating that the drugs do not act through a bypass mechanism. Additional analyses highlighted the large mitochondrial rRNA of the mitoribosome as the primary target of the effective antibiotics, disturbing mitochondrial protein synthesis and determining an unbalanced production of the OXPHOS subunits. In particular, differential effects on the mitochondrial synthesis of the various OXPHOS subunits were observed, leading to a stronger impairment in complex IV assembly. Genetic analyses confirmed the large mt-rRNA of the mitoribosome as the antibiotic target since the absence of the auxiliary factors Rrf1 or Mif3, which are involved in mitochondrial ribosome recycling or in the translation initiation step, resulted in unbalanced mitochondrial protein synthesis and was also able to ameliorate the OXPHOS defect of *bcs1* mutants [28].

Yeast *bcs1* mutants, possibly due to a slowed-down assembly, show an accumulation of inactive pre-complex III_2_ (Pre-III_2_) that can interact with complex IV and give rise to unfunctional supercomplex Pre-III_2_-IV. Treatment with specific concentrations of the antibiotics identified in the screening can dysregulate mitochondrial translation; this is supposed to be beneficial because the decrease in complex IV levels could partially prevent the trapping of Pre-III_2_, allowing the assembly process towards the fully assembled complex III and then the supercomplex III_2_-IV. It must be underlined that this compensatory mechanism could be beneficial only in the case of fine-tuning complex IV reduction; the decrease should be enough to allow at least some Pre-III to proceed toward assembly, but, in order to obtain enough active supercomplex III_2_-IV, a sufficient amount of cIV should be retained and associated with fully assembled III_2_. This could be obtained by deleting *COX8*, which codes for a non-essential complex IV subunit, leading to a 30% decrease in cytochrome aa_3_ [28].

In humans, similar to in yeast, pre-complex III accumulates in patients and interacts with complex IV [110], preventing the formation of the correct supercomplex I-III_2_-IV. The binding of pentamidine, whose binding site is conserved from yeast to humans [111], could decrease both complex I and complex IV levels, as demonstrated in the nematode *C. elegans*, allowing the formation of some functional complex III and supercomplex I-III_2_-IV [28].

### 3.4. Identification of Molecules for the Treatment of OPA1-Related Disorders

OPA1 encodes a mitochondrial dynamin-like GTPase, a multifunctional protein involved in mitochondrial dynamics, including fusion, structural organization of *cristae*, mtDNA stability, assembly of the respiratory supercomplexes, apoptosis, and mitophagy [112,113,114,115,116,117,118]. Mutations in this gene determine DOA (dominant optic atrophy) [119,120], a more severe pathology called DOA-plus (DOA and mitochondrial myopathy with multiple deletions occurring in the mtDNA) [121,122,123], Behr syndrome, Leigh syndrome, and some common neurodegenerative disorders, such as Parkinsonism, dementia, and multiple sclerosis [124,125,126,127,128,129,130]. DOA (OMIM #165500) is an autosomal dominant disorder characterized by an early childhood visual impairment with moderate to severe loss of visual acuity, temporal optic disc pallor, color vision deficits, and a centrocecal scotoma of variable density [131]. Usually, mutations leading to DOA determine the production of a truncated protein, meaning that haploinsufficiency is the genetic mechanism underlying the pathology. DOA-plus (OMIM #125250) is an autosomal dominant disorder characterized by DOA features and complications that lead to a more severe outcome; in particular, sensorineural deafness, ataxia, myopathy, chronic progressive external ophthalmoplegia, peripheral neuropathy, multiple sclerosis-like, and parkinsonism-dementia are present [121,124,132,133]. Point mutations are the main cause of the disease and usually occur in the GTPase domain, indicating a dominant-negative effect as the genetic mechanism underlying the pathology [121,133]. Behr syndrome (OMIM #210000) is an autosomal recessive disorder characterized by early-onset optic atrophy accompanied by neurologic features, including ataxia, pyramidal signs, spasticity, and mental retardation [134,135]. Despite promising results emerging, such as the idebenone treatment [136,137] or genetic engineering to correct OPA1 mutations [138], no approved treatments are currently available for OPA1-related diseases.

The yeast *S. cerevisiae* can be exploited due to the presence of *MGM1*, the ortholog of OPA1, which is involved in mitochondrial morphology [139,140] and in mtDNA maintenance [141,142,143]. Similarly to OPA1, the presence of a functioning Mgm1 protein is necessary for mitochondrial fusion and the maintenance of *cristae* structure [139,144,145,146]. In particular, the yeast strain carrying the hypomorphic mutation p.Ile322Met (*mgm1^I322M^*), equivalent to the human p.Ile382Met variant [147], related to the DOA phenotype, has been exploited, thanks to its thermosensitive phenotype, for the identification of potential therapeutic molecules. Due to the intimate relations between mitochondrial dynamics and mtDNA maintenance, yeast strains carrying *mgm1* pathogenic alleles show a strong increase in *petite* mutants that arise spontaneously after large deletions or loss of mtDNA, giving rise to respiratory deficiency [148]. The *petite* phenotype is an irreversible condition; thus, a thermosensitive mutation should be used in the attempt to identify possible beneficial molecules. The *mgm1^I322M^* strain showed a reduction in oxidative growth using an ethanol or glycerol carbon source at 37 °C. Two different libraries were tested through the drug drop test. Notably, the first library was the Selleck FDA-approved chemical library, for which a drug repurposing approach leads to a potential direct use on patients; the second was the NCI BioServices Diversity Set IV, which collects 1596 molecules with different structural properties, increasing the probability of positive molecule identification, albeit a direct use in clinics is not possible. In total, 22 molecules from the first library and 21 from the second were identified. One molecule, benzbromarone, belonging to both libraries, has been identified as positive in both cases, demonstrating the reproducibility of this test [29]. In order to select the best drug among the 42 positive hits, a sequential step-by-step experimental procedure aimed at skimming the less promising molecules and focusing on the few ones with high translational potential was performed. The analyses of the pharmacological properties and toxicity allowed us to eliminate five anti-tumor compounds that were not suitable for the treatment of mitochondrial pathologies [29]. The positive hits were then tested on another OPA1-disease model. Due to the low similarity between Mgm1 and OPA1 sequences, few pathological mutations can be studied by homologous complementation, and heterologous complementation cannot be pursued as the expression of human cDNA does not complement the absence of the yeast gene. For this reason, another approach was successfully attempted, i.e., the creation of the *MGM1*/OPA1 chimeric gene (called *CHIM3,* encoding the N-terminal region of Mgm1 and the whole GTPase, middle, and GED domains of OPA1) [149]. A variety of pathological mutations were introduced in this chimeric gene to select a suitable mutant for the drug drop test analyses, in particular the mutant harboring the p.Ser646Leu allele, characterized by a strong thermo-sensitive growth impairment. This further step reduced the molecules to twenty-six: fifteen compounds from the Selleck FDA-approved chemical library and eleven compounds from the NCI BioServices Diversity Set IV were able to partially restore the growth of the *chim3^S646^*^L^ strain. Between the latter 11 molecules, only benzbromarone, also identified in the other library, and ovalicin were considered for the subsequent characterization because the other molecules, for various reasons such as cytotoxicity, toxicity, instability in aqueous solutions, and unavailability, were not suitable for the treatment of mitochondrial diseases [29]. The next step was the evaluation of the efficacy of the drugs on mtDNA maintenance. In fact, mutations in *MGM1* alter mtDNA stability, resulting in a *petite* frequency increase; therefore, the molecules identified through the screening were tested to assess whether the rescue of the oxidative growth defect may be due to an increase in mtDNA stability. The results allowed us to further restrict the number of molecules to be tested in higher models. In particular, six molecules (benzbromarone, chloroxine, retapamulin, salicylanilide, tolfenamic acid, and ovalicin) reduced the mitochondrial DNA instability of the *mgm1^I322M^* strain. To validate the effectiveness of these molecules on mammal models, OPA1-disease cell models, such as Opa1^−/−^ mouse embryo fibroblasts (MEFs), expressing the human OPA1 isoform carrying p.Arg445His or p.Asp603His, associated with DOA-plus and DOA, respectively, and patients’ fibroblasts carrying the same mutations, were used. Specifically, five of the six molecules identified through yeast-based screening were also beneficial in MEFs (benzbromarone, chloroxine, tolfenamic acid, salicylanilide, and ovalicin), highlighting tolfenamic acid as the most promising molecule to be used for drug repurposing in a clinical trial for DOA or other neurodegenerative pathologies associated with mutations in the OPA1 gene [29]. Cellular analyses aimed at investigating the molecular mechanisms ruled out a direct involvement of mitochondrial biogenesis and shaped protein expression as the major player in the rescuing effect. A possible common mechanism could be the activation of autophagy. Notably, each drug could have different rescuing mechanisms; in fact, the various defects induced by OPA1 mutations, including mitochondrial network morphology, ATP content, cell viability, or energetics, can be recovered by different drugs.

Interestingly, in order to identify molecules able to suppress mitochondrial fusion and mtDNA maintenance defects, a drug drop test was also performed on the fission yeast *Schizosaccharomyces pombe*, carrying the p.Pro300Ser variant in the *msp1* gene (*msp1^P300S^*), the ortholog of OPA1. About 1600 compounds were tested, and five of them (vanoxerine, hexestrol, clomifene, ketoconazole, and terconazole) prevented the lethality associated with the loss of mtDNA maintenance. This highlights the usefulness of the test, which can also be tailored to organisms other than *S. cerevisiae*, in identifying new drugs for mitochondrial dysfunction rescue [150]. Two compounds, hexestrol and clomifene, suppressed the mtDNA depletion induced by the loss of Msp1p; hexestrol was also beneficial for mitochondrial fragmentation. Data obtained suggest that hexestrol may counteract the effects of the inactivation of mitochondrial fusion, whereas clomifene may modify mitochondrial membrane organization by acting on ergosterol metabolism, which in turn may affect nucleoid anchoring [150]. Although these drugs were not validated using higher eucaryotic OPA1-model organisms, they are already prescribed in humans, and bioavailability and toxicity data are available; hence, they could be potentially repositioned for OPA1-associated diseases.

### 3.5. Identification of Molecules for the Treatment of PANK2-Related Disorders

PKAN (Pantothenate kinase-associated neurodegeneration, OMIM #234200) belongs to the group of rare neurodegenerative diseases with brain iron accumulation (NBIA), characterized by a progressive iron accumulation in the basal ganglia and other areas of the brain. The consequences are extrapyramidal movements, such as parkinsonism and dystonia, along with pigmentary retinopathy and a classic MRI signature, the so-called ‘eye of the tiger’ [151]. A huge variability is present in the age of onset, the severity, and the cognitive involvement (reviewed in [152]). PKAN can be classified into two forms: the classic form is characterized by early onset and a rapid progression of the disease; the atypical form arises instead in the second decade of life with a slower progression [151]. In addition, an intermediate form exists, characterized by early onset and slow progression or later onset and rapid progression. Patients presenting with early onset are inclined to develop pigmentary retinopathy, although patients presenting with later onset usually have speech disorders and psychiatric features [153,154]. Another disease, the HARP syndrome (hypoprebetalipoproteinemia, acanthocytosis, retinitis pigmentosa, and pallidal degeneration), is a less severe autosomal recessive disease characterized by orofacial dyskinesia, dystonia, and progressive dementia. The analyses on an 11-year-old patient showed hypoprebetalipoproteinemia, acanthocytosis, atypical retinitis pigmentosa, iron accumulation in the pallidal nuclei, and abnormal serum lipoproteins [155].

Both PKAN and HARP syndrome are caused by mutations in the PANK2 gene, which encodes the pantothenate kinase, an essential regulatory enzyme involved in the first and limiting step in coenzyme A (CoA) biosynthesis and catalyzes the cytosolic phosphorylation of vitamin B5 (pantothenate), N-pantothenoylcysteine, and pantetheine [156]. Four isoforms of the PANK gene are present in mammals (PANK1, PANK2, PANK3, and PANK4), but only mutations in isoform 2 (PANK2) are related to PKAN and HARP phenotypes. PANK2 is ubiquitously expressed, with a higher expression in the brain [156], and is localized in the mitochondrial inner membrane space [157,158,159] and perhaps in the nucleus [160]. To date, no effective therapeutic solution is available for PKAN, despite symptomatic treatments [161]. Several potential treatments have been proposed (using panthethine, CoA, acetyl-40-phosphopantetheine, fosmetpantotenate, pantothenate, and 40-phosphopantetheine) in light of their effectiveness on different PKAN models [162,163,164,165,166,167,168]. The oral administration of deferiprone in PKAN patients, which showed a reduction in brain iron and a slower progression of the disease, is a promising alternative [169].

Despite the lack of a neural system, *S. cerevisiae* has been successfully used to understand the molecular mechanisms underpinning several neurodegenerative diseases as well as iron-related disorders [170]. Yeast can be exploited as a PKAN model thanks to the presence of a PANK2 ortholog, called *CAB1*. *CAB1* encodes for the unique pantothenate kinase in yeast and co-localizes in the cytoplasm and in mitochondria, with a predominance in the cytoplasm [30]. Ceccatelli Berti and collaborators expressed the PANK2 cDNA in yeast, but the human gene does not complement the *cab1* deletion. Therefore, a homologous approach was used, exploiting the yeast *CAB1* gene that was mutagenized to mimic the human variants identified in patients and introduced into the *cab1Δ* strain through plasmid shuffling [30]. The yeast model recapitulates the main defects found in patients: mitochondrial dysfunction, altered lipid metabolism, iron overload, and oxidative damage. More than 150 variants, including point mutations and frameshift mutations in the PANK2 gene, have been identified through the years; the variants fall in the active site, in the dimerization interface, or in the protein interior [171,172]. The yeast strain carrying the p.Gly351Ser variant (*cab1^G351S^*) acts as a hypomorphic allele and presents a thermosensitive phenotype [173]. Thanks to these features, a drug drop test was performed to evaluate the potential beneficial effects of 1018 FDA-approved compounds [30]. Eight compounds were able to rescue the oxidative growth phenotype of this strain; subsequently, these molecules were tested on the *cab1^N290I^* strain, mimicking the human pathogenic mutation p.Asn500Ile. Four molecules were able to restore the oxidative growth of this mutant, and the two compounds showing the better effect, 5,7 dichloro-8 hydroxyquinoline (CQCl) and nalidixic acid (nalH), were subjected to further studies to determine their rescue ability on other phenotypes due to CoA biosynthesis impairment. In particular, both molecules were able to rescue lipid peroxidation and the iron and ROS content, probably due to their ability to restore the physiological iron content. The authors speculate that the rescue ability could depend on the chelating properties of both CQCl and nalH, leading to excess iron chelation, a consequent reduction in oxidative stress, and maybe membrane damage. Moreover, nalidixic acid also showed a beneficial effect on other mutants (*cab1^S237N^*, *cab1^I287T^*, *cab1^I291T^*, *cab1^I294V^*, and *cab1^A352T^,* mimicking human mutations p.Ser471Asn, p.Ile497Thr, p.Ile501Thr, p.Ile504Val, and p.Ala562Thr, respectively), expanding its potential administration to a wider group of patients [30]. No studies have been performed on more complex models yet; nevertheless, they could be considered potential pharmacological treatments for PANK-related diseases.

### 3.6. Identification of Molecules for the Treatment of POLG-Related Disorders

The DNA polymerase γ, encoded by POLG, is the catalytic subunit of the only mitochondrial replicase present in animal mitochondria [174,175]. POLG interacts with two accessory subunits, encoded by POLG2, that ensure the high efficiency of the complex [176]. More than 300 mutations in POLG have been reported through the years and represent the main cause of mitochondrial diseases with Mendelian inheritance [177]. Several patients harbor three or more mutations and SNPs in this gene, leading to a difficult genetic diagnosis and dissection of the relative contribution of each variant to the pathological outcome. Most POLG mutations lead to multiple deletions or depletion of mtDNA, both of which result in impaired energy production through oxidative phosphorylation. Patients presenting multiple mtDNA deletions show adolescence to adulthood onset, with the involvement of a limited number of tissues and organs, whereas patients with mtDNA depletion present infancy to childhood onset, with several tissues and organs affected (reviewed by [177]). MEMSA (myoclonic epilepsy myopathy sensory ataxia), SCAE (spinocerebellar ataxia with epilepsy, OMIM #607459), MELAS (mitochondrial encephalomyopathy, lactic acidosis, and stroke-like episodes, OMIM #540000), MERRF (myoclonic epilepsy with ragged red fibers, OMIM #545000), and SANDO (sensory ataxic neuropathy, dysarthria, and ophthalmoparesis, OMIM #607459), characterized by epilepsy and ataxia, are the most common syndromes associated with mtDNA deletions [178,179,180,181]. Other diseases are associated with mtDNA depletion caused by POLG mutation, among which are Alpers-Huttenlocher syndrome (AHS; OMIM #203700), characterized by severe encephalopathy with liver failure and intractable epilepsy [182], lethal childhood myocerebrohepatopathy (MCHS), where development delay, myopathy, hepatic failure, pancreatitis, and acidosis are the main features [183], and a MNGIE-like disease (OMIM #613662), characterized by gastrointestinal dysmotility, ptosis, myopathy, and sensory neuropathy [184]. Moreover, adult-onset PEO is the most frequent mitochondrial pathology presenting mtDNA deletions due to mutations in POLG [185,186]. PEO can present recessive inheritance (arPEO), characterized by progressive weakness of the extraocular muscles, which determines ptosis and ophthalmoparesis, or dominant inheritance (adPEO), characterized by axonal neuropathy, ataxia, depression, parkinsonism, and hypogonadism [187]. Substitutions causing adPEO occur predominantly in the polymerase domain, while recessive mutations occur in the whole protein.

*MIP1*, encoding the mitochondrial polymerase, is the yeast *S. cerevisiae* ortholog of POLG [188]. Yeast has been exploited through the years to validate in vivo the pathogenicity of POLG mutations, determine the inheritance pattern (dominant or recessive), dissect the pathological mechanism underlying the mtDNA instability (reviewed by [189]), and, more recently, identify potential therapeutic molecules [31]. In fact, despite the high number of affected patients compared to other mitochondrial diseases, an effective treatment does not even exist for POLG-related disorders. In particular, the strain carrying the recessive allele *mip1^G651S^*, harboring a mutation in the polymerase domain equivalent to the human p.Gly848Ser one, presents a thermosensitive phenotype, resulting in an oxidative growth defect on ethanol at 37 °C, and thus it was exploited to perform a drug drop test screening. A total of 1500 molecules from the TEBU-BIO and Prestwick chemical libraries were tested. Of the total, 30 drugs rescued the oxidative growth defect, and among them, Clofilium tosylate (CLO), an anti-arrhythmic drug presenting low toxicity and showing a strong and dose-dependent rescue of oxidative growth, was chosen to investigate its effect. CLO showed its ability to significantly reduce the *petite* frequency, a hallmark of mtDNA instability, of eleven different mutant strains. The mutations in these strains affect residues localized throughout the different Mip1 domains, thus suggesting a mutation-independent beneficial effect of the treatment. It should be noted that the more effective the molecule, the less the mtDNA stability in the mutant was impaired. Additionally, western blot results showed that CLO supplementation stabilizes Mip1 protein, increasing the amount of a mutant protein characterized by partial retention of catalytic activity. This increase could compensate, at least partially, for the defective polymerase activity, possibly by increasing mtDNA replication [31]. Despite the fact that a direct action of CLO on mitochondrial membrane potential, polymerase fidelity, the mitochondrial recombination process, and the dNTP pool were ruled out, the target of this drug is still unknown.

The promising results obtained in yeast led to studies on different model systems, such as *C. elegans*, patients’ fibroblasts, and zebrafish. In particular, CLO was able to rescue a *C. elegans*-specific defect, namely gonadal function compromise, which leads to sterility. Studies on *C. elegans* of two additional molecules, ibutilide and dofetilide, characterized by a chemical structure similar to CLO, allowed the animals to reach adulthood but showed less efficacy relative to CLO. The fibroblasts were derived from a patient with compound heterozygous POLG mutations located in the polymerase domain, leading to severe mtDNA depletion. In a quiescent experimental condition, CLO treatment led to a significant increase in mtDNA copy number and an increased POLG protein level, as observed in yeast. In zebrafish, supplementation with CLO improved the detrimental phenotypes observed in organisms harboring a homozygous hypomorphic non-lethal point mutation in polg. This results in an increase in mtDNA levels and complex I activity, as well as the rescue of the myofibril organization and the mitochondrial mass. Cardiac phenotypes such as atrial enlargement and tachycardia, induced by morpholino-mediated Polg deficiency, could be restored to normal values after treatment with CLO [190]. Thus, the biochemical mechanism whereby clofilium tosylate enhances mtDNA replication is conserved between yeast and vertebrates, confirming the usefulness of yeast in discovering new molecules for the treatment of POLG-related diseases [31].

### 3.7. Identification of Molecules for the Treatment of MPV17-Related Disorders

MPV17 encodes a mitochondrial inner membrane protein involved in mitochondrial deoxynucleotide homeostasis and mtDNA maintenance [191]. The MPV17 protein takes part in a high molecular weight complex [192,193], forming a non-selective channel in the IMM (inner mitochondrial membrane) [194]. However, the precise physiological role of the channel and the nature of the cargo remain elusive. The MPV17 protein is also directly or indirectly involved in the maintenance of mitochondrial membrane potential, reactive oxygen species (ROS) generation, and mitochondrial *cristae* formation [192,195,196,197]. Recessive mutations in MPV17 are related to hepatocerebral type 6 mitochondrial DNA depletion syndrome (MDDS) (OMIM #256810), Navajo neuro-hepatopaty (OMIM #256810), and type 2EE Charcot Marie Tooth disease (OMIM #618400) [194,198]. The clinical features associated with MPV17 mutations are highly variable, but hepatopathy and neurological abnormalities are the most frequent presentations [199]. The manifestations commonly occur in the first months of life or in infancy, despite cases of adult-onset leukoencephalopathy, PEO, or neuropathy that are characterized by multiple mtDNA deletions [200,201,202]. The hepatocerebral type 6 MDDS, which belongs to a group of diseases characterized by mtDNA depletion due to defects in replication or in mitochondrial dNTP homeostasis, is characterized by an infantile onset of progressive liver failure, often leading to death in the first year of life. A progressive neurologic involvement usually affects patients that survive, including ataxia, hypotonia, dystonia, and psychomotor regression [203]. Navajo neuro-hepatopathy was prevalent in the Navajo population of the southwestern United States. The patients present with liver disease, severe sensory and motor neuropathy, corneal anesthesia and scarring, cerebral leukoencephalopathy, failure to thrive, and recurrent metabolic acidosis with intercurrent illness [198,204]. The similarities in clinical, pathological, and biochemical features between patients affected by Navajo neurohepatopathy and by MDDS suggested abnormal regulation of mtDNA copy number as the primary cause of the disease [205]. Type 2EE Charcot Marie Tooth disease is an autosomal recessive sensorimotor peripheral axonal neuropathy with onset in the first or second decades of life. The disorder primarily affects the lower limbs and is slowly progressive, sometimes resulting in loss of ambulation with a later onset of upper limb involvement. There is significant distal muscle weakness and atrophy, usually with foot or hand deformities (summarized by [191]). Treatment is limited to nutritional support to prevent hypoglycemia and requires frequent feeds, especially with uncooked cornstarch. Liver transplantation remains the only treatment option for liver failure, but it is controversial because of the multisystem involvement in this disorder [206], so it could not significantly alter the progression of the disease.

*SYM1* (Stress-inducible Yeast MPV17) was identified in 2004 as the yeast ortholog of MPV17; it is induced by heat stress and is necessary for growth on ethanol at 37 °C [204,207]. Due to the functional conservation between yeast and human genes, yeast has been exploited both to validate the alleged pathological mutations identified in patients and to attempt to elucidate the puzzling and elusive Sym1/MPV17 protein function [32,192,194,195,208]. Interestingly, all the yeast mutant strains carrying pathological mutations showed mtDNA instability, as demonstrated by the increased *petite* frequency [194,208].

As no effective treatment exists [18], yeast *S. cerevisiae* has been used to identify new potential molecules for the treatment of MPV17-related disorders; in particular, the *sym1^R51W^* strain was used to perform a drug drop test with the Selleck Chemicals FDA-approved library. The strain showed a reduction in oxidative growth on ethanol at 37 °C, which was rescued by eleven molecules. Moreover, six additional compounds belonging to the Prestwick and TEBU-BIO chemical libraries were tested, and one of them, imazalil, showed beneficial effects. Interestingly, half of the twelve molecules have the ergosterol pathway as a common target; therefore, only three of them were subjected to further investigations. By testing the potential beneficial effect on the *sym1Δ* strain in the drug drop test-like screening, all the molecules were positive, suggesting a bypass mechanism of action [32]. The null mutant strain and several strains carrying the equivalent mutations to those reported in patients showed an increased *petite* frequency [194,208]. Furthermore, the null mutant is characterized by a decrease in mitochondrial dNTPs, strongly suggesting that mtDNA instability in Sym1-deficient cells is due to a shortage of precursors for DNA synthesis, as also hypothesized in zebrafish, Mpv17^−/−^ mice, and MPV17-deficient human fibroblasts [209,210,211]. This underlies the fact that the “pathogenic” mechanism of Sym1/MPV17 dysfunction, i.e., the decrease in dNTP levels leading to mtDNA depletion/deletion, is conserved from yeast to humans.

To get insight into the molecular effect of the beneficial molecules, their ability to rescue mtDNA instability and decrease dNTPs was measured. All the molecules determined a reduction in the *petite* frequency in the *sym1^R51W^* and *sym1Δ* strains, confirming their rescuing activity of mtDNA instability, and an increase in the mitochondrial dTTP pool in *sym1∆* [32].

The fact that half of the identified drugs modulate ergosterol biosynthesis suggests a beneficial effect by increasing the permeability of mitochondrial membranes. In mammalian cells, the ergosterol counterpart is cholesterol; hence, its moderate reduction could have beneficial effects in MPV17-related disorders. Since the cholesterol pathway is crucial for several cellular functions, it should be carefully modulated, possibly with no influence on collateral pathways such as the mevalonate pathway. For this reason, statins, the most commonly used cholesterol-lowering drugs, are not suitable; specific inhibitors of CYP51A1 (lanosterol 14-α-demethylase) could instead be a better target. Since antimycotic inhibitors of ergosterol synthesis have low activity on human CYP51A, other molecules should be considered.

In addition to MPV17, mitochondrial DNA depletion syndromes (MDDS) are caused by mutations in several nuclear genes, including ABAT, AGK, DGUOK, MGME1, POLG, RNASEH, RRM2B, SUCLA2, SUCLG1, TFAM, TK2, TYMP, and TWNK, which are involved either in mtDNA replication or in the maintenance of a balanced mitochondrial nucleotide pool (reviewed in [212,213]). MDDS are a group of autosomal recessive disorders with a wide genetic and clinical spectrum and are characterized by a severe reduction in mtDNA content in affected tissues and organs, leading to organ dysfunction.

To date, only 100 affected individuals have been identified with MPV17-related mtDNA maintenance defects. In light of this restricted number of patients, to evaluate whether the drugs may be beneficial for other mutations associated with MDDS and thus expand their potential therapeutic use, they were tested on two further yeast models: *RNR2*/RRM2B and *MIP1*/POLG. In these models, an increase in the dNTP pool was shown to be beneficial for the maintenance of the mtDNA [32,214].

*RNR2,* the yeast ortholog of RRM2B, encoding the small p53-inducible ribonucleotide-diphosphate reductase, is involved in DNA repair and mtDNA synthesis in post-mitotic cells [215]; mutations in RRM2B were found in patients affected by MDDS types 8A and 8B (OMIM #612075). The pathogenic mechanism in RRM2B deficiency is due to the decrease in the dNTP pool caused by faulty ribonucleotide reductase activity, leading to mtDNA depletion/deletion [216]. Several pathogenic RRM2B variants were introduced in *S. cerevisiae* by the homologous complementation approach. The Leu362Val substitution in yeast *RNR2*, equivalent to the human Leu317Val, showed the highest mtDNA maintenance defect and was thus used to test the *SYM1*-beneficial molecules. All the molecules, except Sertraline, determined a significant reduction in *petite* frequency, similar to that observed by overexpressing the *RNR1* or *RNR4* genes, which encode the large and small subunits of the RNR complex. Thus, increased levels of dNTPs (both drug- and genetically-induced) could prevent mtDNA instability and represent a potential target for the treatment of RRM2B-related pathologies [32].

*MIP1*, an ortholog of the human POLG, encoding the catalytic subunit of mtDNA polymerase γ, is involved in mtDNA replication, and its role in mitochondrial dysfunction was presented in the previous paragraph. The pathogenic mechanism in POLG deficiency is due to faulty polymerase activity, leading to mtDNA depletion/deletion. It was demonstrated in yeast that the mtDNA defects associated with *mip1^G651S^*, equivalent to the human POLG p.Gly848Ser pathogenic variants, one of the most common POLG mutations described previously, could be corrected by increasing the amount of dNTPs, the substrates of the enzyme [214]. Six *SYM1*-beneficial molecules were able to reduce the *petite* frequency of the *mip1^G651S^* strain [32]. This result has huge translational potential since POLG mutations are the most common cause of mitochondrial epilepsy and have been correlated with 10–25% of progressive external ophthalmoplegia (PEO) and >10% of ataxia cases [177].

In the case of MDDS due to impairment of the mtDNA synthesis machinery, such as for POLG-related diseases, or due to nucleotide metabolism, such as for RRM2B and MPV17-related diseases, a proposed therapeutic option is the supplementation of deoxyribonucleosides [210,217,218,219]. The work by di Punzio and coworkers shows an alternative strategy based on drugs acting directly or indirectly on the dNTP synthesis pathway or on mitochondrial import, thus increasing the intramitochondrial dNTP pools [32]. It is possible to speculate that such a kind of modulation might also positively act on MDDS due to mutations in other genes. Although, on one side, the molecular mechanism of action of the identified drugs is not clear and further investigations are required to demonstrate their efficacy on multiorgan eukaryotic models, on the other side, the drugs are FDA-approved and thus may be repurposed for MDDS treatment.

### 3.8. Identification of Molecules for the Treatment of TAFAZZIN-Related Disorders

Barth syndrome (BTHS; OMIM #302060), also known as 3-methylglutaconic aciduria type II, is a recessive X-linked disorder caused by mutations in the TAFAZZIN gene. This pathology, first described by Barth and collaborators [220], is characterized by dilated cardiomyopathy with endocardial fibroelastosis (EFE), a predominantly proximal skeletal myopathy, growth retardation, neutropenia, and organic aciduria, particularly an excess of 3-methylglutaconic acid. In addition, other symptoms can occur, including hypertrophic cardiomyopathy, isolated left ventricular noncompaction, ventricular arrhythmia, motor delay, poor appetite, fatigue, exercise intolerance, hypoglycemia, lactic acidosis, hyperammonemia, and dramatic late catch-up growth after growth delay throughout childhood [221]. BTHS patients present mitochondria with reduced levels of cardiolipin (CL), an acidic diglycerophospholipid prevalently localized in the mitochondrial inner membrane; increased levels of monolysocardiolipin; abnormal ultrastructure; respiratory activity deficiencies; increased ROS content; reduced capacity to sustain apoptosis; and increased cell proliferation [222,223,224,225,226,227,228,229,230,231]. The TAFAZZIN gene encodes a mitochondrial transacylase involved in cardiolipin remodeling. Mutations in this gene determine the accumulation of cardiolipin with incomplete fatty acid chains, leading to multiple mitochondrial alterations that result in respiratory activity impairment [227,228,229,230,231].

The yeast *S. cerevisiae* has been exploited through the years to study the synthesis and remodeling of cardiolipin and, therefore, how mitochondria are influenced by the misregulation of these processes [232,233,234,235]. *TAZ1* is the yeast ortholog of TAFAZZIN and encodes the lysophosphatidylcholine acyltransferase required for the normal phospholipid content of mitochondrial membranes, particularly in regulating cardiolipin content. The yeast strain presenting the deletion of *TAZ1* (*taz1Δ*) showed monolysocardiolipin accumulation and low levels of cardiolipin [232,236,237,238] and is characterized by a respiratory defect at elevated temperatures [236,237,239,240,241]. De Taffin de Tilques and collaborators, through experiments on the *taz1Δ* yeast strain, identified *ODC1* (the mitochondrial oxodicarboxylate carrier) as a potential therapeutic target for several mitochondrial diseases, including BTHS [33]. Oleic acid was reported to stimulate *ODC1* expression [66] and was thus screened in a drug drop test-like experiment to evaluate its ability to suppress the respiratory defect of *taz1Δ*. After incubation at 36 °C on an ethanol-containing medium, a halo of enhanced growth around the filter was visible, demonstrating the beneficial effect of this molecule and its potential use for the treatment of tafazzin-related disorders.

Furthermore, using a drug drop test-like technique on the *taz1Δ* mutant strain, cytosolic protein synthesis inhibitors showed their ability to rescue the oxidative growth phenotype; in particular, cycloheximide, anisomycin, and emetine, which were rationally selected and tested, suppressed the oxidative defect in a dose-dependent manner. Cycloheximide was beneficial at concentrations 20–30 times lower than the other protein synthesis inhibitors tested and determined the best growth improvement, making it the most interesting compound [34]. Therefore, studies in yeast prove that partial inhibition of cytosolic protein synthesis preserves both oxidative phosphorylation and mtDNA maintenance. To confirm the yeast data, experiments on HeLa cells deficient in tafazzin have shown that low concentrations of cycloheximide significantly improved the proliferative capacity. Together, the results obtained in yeast and the HeLa cell model showed that CL remodeling deficiency presents with similar phenotypes and demonstrated that cytosolic protein synthesis is a potential therapeutic target for alleviating these defects and treating Barth syndrome [34].

Interestingly, *ODC1*, and thus the transport of oxodicarboxylic acids across the IMM, and modulation of cytosolic protein synthesis were identified as potential targets for the treatment of other mitochondrial diseases (i.e., ATP synthase assembly defects, MPV17-related MDDS, ANT1-related adPEO [67,192,242]), allowing us to speculate on a possible common therapeutic approach for a large spectrum of mitochondrial diseases.

## 4. Discussion

Mitochondrial disorders (MDs) are a group of rare diseases with a prevalence of approximately 1.6 in 5000 live births, characterized by progressive and incurable defects that can result in premature death. The genetic, clinical, biochemical, and molecular heterogeneity of MDs and the limited availability of preclinical models have strongly hampered the development of effective therapeutic interventions. In fact, there is no resolutive treatment for almost any MD, and current therapeutic management is predominantly focused on relieving symptoms and treating complications. These actions are important to improve the quality of life of patients and their families; however, these conditions are usually progressive, leading to major disabilities. Even if, in recent decades, significant progress has been made (reviewed in [243]), pharmacological treatment is still inadequate. The identification of molecules with positive effects on mitochondrial functions in experimental models could be the first step toward pharmacological therapies. In this context, despite its simplicity, the yeast *S. cerevisiae* can be considered a powerful ally both for modeling mitochondrial disorders and for searching for beneficial drugs by means of the drug drop test method. In fact, thanks to all the advantages discussed in the introduction, such as the flexibility of genetic tools combined with the high conservation of protein sequence and function, this organism can be considered an excellent “mitochondrial disease model”. In particular, both nDNA and mtDNA mutations can be easily reproduced in *S. cerevisiae* thanks to efficient homologous recombination and plasmid transformation, or biolistic transformation. Over the years, a variety of yeast disease models have been produced (reviewed in [244,245,246,247,248]), allowing validation of alleged pathological variants (i.e., demonstrating the causal relationship between mutation and disease) and insight into the molecular mechanisms underlying the clinical manifestation. More recently, yeast has also demonstrated itself to be a valuable tool for the identification of new therapeutic interventions. To this purpose, the drug drop test, an easy phenotype-based high-throughput method, was developed, leading to the possibility of performing screenings of thousands of compounds in a cost-effective and time-saving manner [23]. Furthermore, by using FDA-approved compounds for which toxicity and bioavailability studies have been carried out in humans, a repurposing approach could be exploited, thus speeding up the usually very slow drug discovery process. This method is useful not only for mitochondrial diseases, as described in the present review, but is also very versatile and lends itself to being easily adapted to other contexts. For example, an adapted drug drop test based on the addition of glucose and SDS to the solid medium instead of a non-fermentable carbon source was used to find new compounds and drug targets for VPS13-dependent neurodegenerative diseases (reviewed by [24]), allowing the identification of calcineurin inhibitors [249], flavonoids [250], and copper ionophores [251] to suppress the *vps13Δ* phenotype. Binieda and colleagues exploited yeast to perform a drug screening to identify compounds active against the Charcot Marie Tooth-GDAP1 model; in particular, an adapted drug drop test using a Ca^2+^-containing medium on the *csg2Δ* mutant allowed the identification of two active compounds [25]. Yeast and an adapted drug drop test have also been exploited to identify doxorubicin and analog compounds able to interfere with the immune evasion by Epstein-Barr virus [252].

Regarding MDs, the drug drop test method was successfully applied to several yeast disease models, leading to the identification of many molecules (summarized in Table 2), and the results obtained are very promising, confirming the usefulness of this system. In particular, the most relevant aspect is that several molecules identified in yeast have been tested and proved to be effective in more complex models (e.g., *C. elegans*, zebrafish, patients’ fibroblasts) (Table 2), demonstrating the conservation of these therapeutic effects. The positive effects are thus not yeast-specific and are also expected to be valid for humans.

Of course, not all the drugs identified could be considered good candidates to be used for long-term administration, which is instead required for mitochondrial pathologies. For example, the use of chemotherapy medications, characterized by high toxicity and lots of side effects, cannot be pursued. Nevertheless, the identification of these types of molecules can be fruitful in discovering potential molecular targets or pathways that can be modulated by non-toxic analogous molecules in order to develop usable and safe medications. Similarly, some drugs could be expected to be inactive in mammals due to the non-conservation of the binding site; this is, for instance, the case with clarithromycin. Again, the identification of this molecule in yeast is not useless, as other rationally selected drugs acting on the same target could be used instead. Additionally, the direct use of azoles, antifungal molecules targeting the ergosterol/cholesterol pathway and inhibiting lanosterol 14-α-demethylase, in patients is not tenable. In fact, despite the degree of conservation between fungal and human enzymes, they have weak inhibitory activity on the human enzyme. It would be interesting to test a synthetic, potent, and stoichiometric human CYP51 inhibitor that was recently identified [253].

In several cases, it was shown that the molecules were active in yeast, although it does not possess the known molecular target of these drugs, suggesting they could act on one or more unknown secondary targets. In fact, it is currently accepted that a drug acts on a multitude of targets in the cell, potentially regulating several different pathways and leading to off-target effects.

The identification of the target/pathway by which the drug can rescue the yeast defect could be of great interest not only from a therapeutical point of view but also because this could help to elucidate the molecular pathogenic mechanism of the disease. Target identification can be addressed using the yeast collection of heterozygous deletants by systematic chemical-genomic profiling. Haploinsufficiency of a heterozygous deletion mutant, pointed out by a marked sensitivity to a compound, indicates that the deleted gene is a molecular target of the chemical [254,255,256,257,258]. This approach, for example, revealed the mitochondrial protein sorting pathway as a potential intervention point for the treatment of ATP synthase deficiency [26]. Nevertheless, although the discovery of the drug target could be very scientifically interesting, phenotype-based screenings enable the identification of active compounds regardless of knowledge about the target and its mechanism of action. For several drugs presented in this review, the specific pathway by which the drug is able to rescue the mutant’s defect was not identified by the authors. However, these drugs could be considered for translational use anyway, especially if the chemicals were also effective in other eukaryotic models.

Considering the rarity of patients affected by a specific mutation, an interesting approach is to look for molecules that are effective for a group of diseases, widening their possible application. Looking at the results obtained on different yeast-disease models, it is possible to notice that some molecules come up independently in different screenings. For example, sertraline was identified both in ANT1-related and MPV17-related screenings; oleic acid was demonstrated to be beneficial in *fmc1Δ* and *taz1Δ* mutants; and nalidixic acid was identified as an active compound for PANK2-related and OPA1-related diseases. This highlights the possibility of cross-reactions and common pathways as possible intervention points. This hypothesis could be directly addressed by testing chemicals that were active in a model on other models, as performed by di Punzio and collaborators. They showed that half of the drugs identified as active on yeast MPV17-disease models are also effective on two other disease models characterized by the same molecular defect, i.e., mtDNA depletion [32].

Interestingly, a variety of chemicals identified are supposed to act directly or indirectly on the membrane composition or the membrane permeability property, highlighting IMM as a major target for MDs treatment.

In addition to the screening of a complete library, a drug drop test can also be used to directly test a small number of rationally selected chemicals, as performed for TAFAZZIN-related diseases [33,34].

## 5. Conclusions

**Figure 2:**
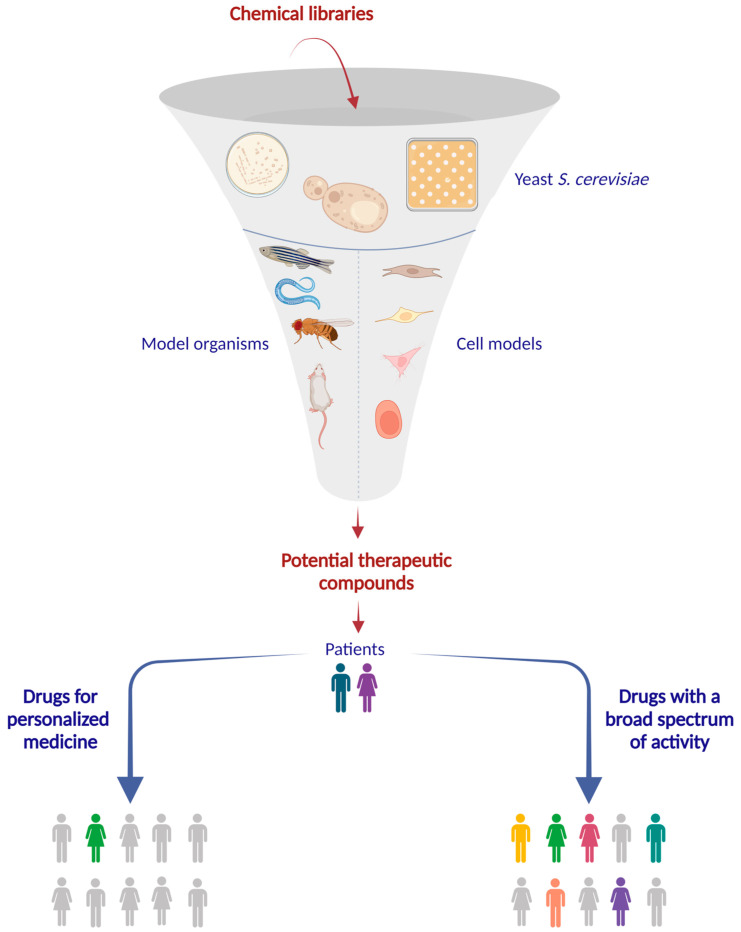
From yeast to patients: the drugs’ path. A huge number of molecules (belonging to chemical libraries) could be tested in yeast. Active compounds could be further analyzed in other model organisms and/or cell models, and each step determines a reduction in the number of potential therapeutic compounds. In the end, the most promising molecules could be administered to patients to treat MDs. Two outcomes could be envisaged: personalized medicine, when a drug is active only on a specific mutation and therefore applicable to a single or few patients; alternatively, some drugs could present a broad spectrum of activity, being suitable for a higher number of patients presenting mutations in different genes. Created with BioRender.com on 22 June 2023.

In the last few years, the yeast *Saccharomyces cerevisiae* has proven to be an invaluable model system to rapidly and economically discover beneficial molecules with high translational potential for the pharmacological treatment of MDs. To date, the phenotype-based high-throughput screening, called the drug drop test, has been successfully carried out for a variety of disease models, leading to very promising results, strengthened by the fact that studies in more complex eukaryotic model organisms, such as *C. elegans* or zebrafish, and studies in cell models, such as patients’ fibroblasts, confirmed the effectiveness of the drugs. The data presented in this review underline the usefulness of yeast screening as a novel approach to revealing new potential therapeutic molecules for MDs. In Figure 2, a possible drug path from yeast to patients is represented. In the future, we could reason to further and continuously expand the yeast disease models exploited for the drug drop test approach and draw up a comprehensive list of positive hits to be tested on a variety of yeast disease models. This approach will strongly benefit from the availability of numerous mutant strains carrying human pathological mutations. Two almost opposite outcomes could be envisaged. The first is the identification of drugs with selective or predominant effects only on specific genetic conditions or even specific pathogenic variants. This could be considered an example of personalized medicine. The second is the identification of drugs with a broad spectrum of activity against several different genetic defects, which may be due to compensatory mechanisms. The so-called “one-size-fits-all” approach could thereby lead to its possible application to a wide cohort of patients with different MDs. Such an approach was recently tested in our laboratory, showing promising results.

## Figures and Tables

**Table 1:** *S. cerevisiae* models in which a drug drop test has been applied to identify new molecules for the treatment of MDs.

Human Gene	Protein Role	Related Diseases	Yeast, *S. cerevisiae* Gene	Yeast Strain Used for Drug Drop Tests	Positive Hits	References
ATP6, ATP12, TMEM70	Complex V subunit or assembly factor	ATP-synthase deficiencies	*FMC1*	*fmc1*Δ	Yes	[23,26]
			*ATP6*	*T8993G*	Yes	[23]
				*T8851C*	Yes	
				*T9176G*	No	
ANT1	Mitochondrial ADP/ATP translocator	adPEO, MDDS	*AAC2*	*aac2^M114P^*	Yes	[27]
				*aac2^A128P^*	Yes	
				*aac2^S303M^*	Yes	
				*aac2^R96H^*	No	
				*aac2^R252G^*	No	
				*aac2Δ*	No	
BCS1L	Complex III assembly factor	Complex III-related disorders	*BCS1*	*bcs1^F342C^*	Yes	[28]
				*bcs1^K192P^*	No	
				*bcs1^F401I^*	Yes	
OPA1	Mitochondrial dynamin-like GTPase	DOA, Behr syndrome, Leigh syndrome, neurodegenerative disorders, MDDS	*MGM1*	*mgm1^I322M^*	Yes	[29]
				*chim3^S646L^*	Yes	
PANK2	Pantothenate kinase	PKAN, HARP syndrome	*CAB1*	*cab1^G351S^*	Yes	[30]
				*cab1^N290I^*	Yes	
POLG	DNA polymerase γ	MDDS and mitochondrial DNA multiple deletion-related pathologies	*MIP1*	*mip1^G651S^*	Yes	[31]
MPV17	Mitochondrial inner membrane protein	MDDS, Navajo nuro-hepatopaty, type 2EE Charcot Marie Tooth disease, arPEO	*SYM1*	*sym1^R51W^*	Yes	[32]
				*sym1*Δ	Yes	
TAFAZZIN	Mitochondrial transacylase	Barth syndrome	*TAZ1*	*taz1*Δ	Yes	[33,34]

**Table 2:** Active compounds identified through drug drop tests.

Molecule	Yeast Model Used for the Identification of the Active Drug	Further Yeast Model(s) in Which the Drug Was Active	Human Ortholog Gene	Additional Eukaryotic Model(s) in which the Drug Was Active	References
Dihydrolipoic acid Sodium pyrithione	*fmc1*Δ		-	Human NARP-cybrids	[23,26]
Oleate/Oleic acid	*fmc1*Δ *taz1*Δ		- TAFAZZIN	Human NARP-cybrids	[23,33]
Chlorhexidine	*fmc1*Δ	*atp6-T8993G* *atp6-T8851C*	- ATP6	Human NARP-cybrids	[23]
Doxorubicin Epirubicin HCl Daunorubicin HCl	*aac2^M114P^*		ANT1		[27]
Trifluoperazine 2HCl Benzydamine HCl	*aac2^M114P^*	*aac2^A128P^* *aac2^S303M^* *AAC2/aac2^M114P^* *AAC2/aac2^R96H^*	ANT1		[27]
Pergolide mesylate	*aac2^M114P^*	*aac2^A128P^* *aac2^S303M^* *AAC2/aac2^M114P^* *AAC2/aac2^R96H^*	ANT1	ant1-1 *C. elegans*	[27]
Otilonium bromide	*aac2^M114P^* *sym1^R51W^*	*aac2^A128P^* *aac2^S303M^* *AAC2/aac2^M114P^* *AAC2/aac2^R96H^* *sym1*Δ *rnr2^L362V^*	ANT1 MPV17 RRM2B	ant1-1 *C. elegans*	[27] [32]
Sertraline HCl	*aac2^M114P^* *sym1^R51W^*	*aac2^A128P^* *aac2^S303M^* *AAC2/aac2^M114P^* *AAC2/aac2^R96H^* *sym1*Δ *mip1^G651S^*	ANT1 MPV17 POLG		[27] [32]
Pentamidine	*bcs1^F342C^*	*bcs1^F401I^*	BCS1L	*C. elegans*	[28]
Clarithromycin	*bcs1^F342C^*	*bcs1^F401I^*	BCS1L		[28]
Benzbromarone Salicylanilide Tolfenamic acid Ovalicin	*mgm1^I322M^*	*chim3^S646L^*	OPA1	MEFs Patients’ fibroblasts	[29]
Bromhexine HCl Clofazimine Diclazuril Ethinyl Estradiol Fesoterodine fumarate Licofelone Lithocholic acid Piromidic acid Retapamulin Terbinafine hydrochloride Several non-FDA-approved drugs *	*mgm1^I322M^*	*chim3^S646L^*	OPA1		[29]
Chloroxine	*mgm1^I322M^*	*chim3^S646L^*	OPA1	MEFs	[29]
Nalidixic acid	*mgm1^I322M^* *cab1^G351S^*	*chim3^S646L^* *cab1^N290I^* *cab1^S237N^* *cab1^I287T^* *cab1^I291T^* *cab1^I294V^* *cab1^A352T^*	OPA1 PANK2		[29] [30]
5,7 dichloro-8 hydroxyquinoline	*cab1^G351S^*	*cab1^N290I^*	PANK2		[30]
Clofilium tosylate	*mip1^G651S^*	*mip1^G259R^* *mip1^C261R^* *mip1^H734Y^* *mip1^A692T^* *mip1^P829L^* *mip1^R467W^* *mip1^G807R^* *MIP1/mip1^E698G^* *MIP1/mip1^K749R^* *MIP1/mip1^Y757C^*	POLG	*C. elegans* Patients’ fibroblasts Zebrafish	[31,190]
Itraconazole Sertraconazole nitrate	*sym1^R51W^*		MPV17		[32]
Posaconazole Fenticonazole nitrate Haloperidol Benzethonium chloride Imazalil	*sym1^R51W^*	*sym1*Δ *rnr2^L362V^* *mip1^G651S^*	MPV17 RRM2B POLG		[32]
Thonzonium bromide Alexidine HCl Domiphen bromide	*sym1^R51W^*	*sym1*Δ *rnr2^L362V^*	MPV17 RRM2B		[32]
Cycloheximide	*taz1*Δ		TAFAZZIN	HeLa cells	[34]
Anisomycin Emetine	*taz1*Δ		TAFAZZIN		[34]
Vanoxerine ^#^ Hexestrol ^#^ Clomifene ^#^ Ketoconazole ^#^ Terconazole ^#^	*msp1^P300S^*	*msp1^WT^*	OPA1		[150]

* Pyridine-2-Azodimethylaniline; 5-Fluoro-7-nitroquinolin-8-ol; 4h-1,3-Thiazine-5,6-dihydro-4,4-dimethyl-2-(2-phenylethene); 1-Carbamimidoyl-3-(4-cholorophenyl) thiourea; 5-Benzyl-4-phenyldihydrofuran-2-3 dione; 1,1,3-Triphenylpropargyl alcohol; 4-Cholorophenylquinoline-6-sulphonate; 5-[(3-Chloroanilino)-1,2,4-dithiazol-3-ylidene]-dimethylammonium HBr; Benzbromarone (3,5-dibromo-4-hydroxyphenyl-2-ethyl-3-benzofuranyl ketone), (methanone, (3,5-dibromo-4-hydroxyphenyl) (2-ethyl-3-benzofuranyl)-); Albacarcin V, chrysomycin A. ^#^ Molecules identified using *S. pombe*.
